# Heredity and Accomplishment

**Published:** 1913-11

**Authors:** 


Heredity and Accomplishment. Frederick Adams Woods, *Pop. Sci. Monthly*, May, 1913, analyses the heredity of the forty-six Americans honored by tablets in the Hall of Fame. Twenty-six had two or more relatives sufficiently prominent to be listed in biographic dictionaries. He holds that the ordinary run of mortals, composing 99 per cent. of the race produce no more men of the highest rank than the 1 per cent. who constitute the cream of the cream, eugenically and with special reference to distinguished connections. Such statements are rank heresy from the standpoint of a people which believes that all men are created free and equal. But, the medical profession is accustomed to seeking the truth, irrespective of theory. There is something to be said on the other side of the matter. A great many very ordinary persons have distinguished, widely separated ancestry. We know two families descended from the Princess Mary, daughter of Henry VII, immortalized in "When Knighthood Was in Flower," and have another friend tracing back to Henry VII in another line. None of these require us to stand in their presence. The chances are that instead of 99 per cent. of the race (at least in America) having no distinguished connection, there are only 20 per cent. lacking in this respect, and perhaps many of these lack definite knowledge of their ancestry rather than the ancestors themselves. What has impressed us strongly is the tremendous saving of time and opportunity for advancement which even a slight amount of wealth or family influence afford the young man, enabling him to accomplish work, affording him means of pushing himself, and giving him openings, which his less fortunate rival cannot, under ordinary circumstances, secure till he has reached a time of life when he feels that it is too late to start on his real, potential career. On the other hand, it must be conceded that men like Lincoln and Grant, who apparently illustrate the American theory of making good on personal merit, usually show distinguished heredity.

In all fairness, there is also another side to the question. A little girl in school, being asked to what circumstance Columbus mainly owed his reputation, replied that it was because none had discovered America before him. While, in the light of the higher criticism of history, it must be admitted that America had been discovered ten or a dozen times previously, her reply was, for practical purposes, correct. There is no longer opportunity for Columbuses, Cabots, Rolandos and Sylviuses, nor Pearys and Vaters. To confine ourselves to our own profession, most of the specific germs have been discovered. Many a man devotes time and skill to a work that turns out negatively, or, at most, throws some sidelight on medical science. Such research denotes as great mental ability and patience as that of the man whose work turns out successfully. They are like peace presidents and generals who never have the chance to rise to historic greatness.

After all, the man who does his work faithfully, who achieves his own best ideals, who serves his fellows well, represents, in the average, something of more value to the race than the man who gets a tablet in the Hall of Fame.

